# Supplementary material for: A UMLS-based spell checker for natural language processing in vaccine safety
Source: BMC Med Inform Decis Mak. 2007 Feb 12;7:3. doi: 10.1186/1472-6947-7-3 (PMC1805499; doi:10.1186/1472-6947-7-3)
Supplement: Additional file 13 — RAP application directory. Sets up directory for RAP (RDF application for PHP) source code [file 1472-6947-7-3-S13.gz › rap/doc/phpdoc/rdql/RdqlEngine.html]

xml version="1.0" encoding="iso-8859-1"?


Docs For Class RdqlEngine


## Class RdqlEngine

Description

Description |
Descendents
| Methods

Some general methods common for RdqlMemEngine and RdqlDbEngine

History:

- access: public
- author: Radoslaw Oldakowski <radol@gmx.de>
- version: V0.9.1

Located in /rdql/RdqlEngine.php (line 20)

```
Object
   |
   --RdqlEngine
```

Direct descendents

Description |
Descendents
| Methods

| Class | Description |
| --- | --- |
| RdqlDbEngine | This class performs as RDQL query on a DbModel. |
| RdqlMemEngine | This class performes as RDQL query on a MemModel. |

Methods

Description |
Descendents |
Methods

#### Inherited Methods


Inherited From Object

> Object::toString()

Documentation generated on Thu, 7 Jul 2005 13:43:13 +0200 by phpDocumentor 1.3.0RC3
